# Determinants of Peak Oxygen Uptake at Each Stage of Renal Dysfunction in Patients with Heart Disease

**DOI:** 10.31083/j.rcm2306191

**Published:** 2022-05-27

**Authors:** Asami Ogura, Kazuhiro P. Izawa, Shinji Sato, Hideto Tawa, Fumie Kureha, Masaaki Wada, Masashi Kanai, Ikko Kubo, Ryohei Yoshikawa, Yuichi Matsuda

**Affiliations:** ^1^Department of Rehabilitation, Sanda City Hospital, 669-1321 Hyogo, Japan; ^2^Department of Public Health, Graduate School of Health Sciences, Kobe University, 654-0142 Hyogo, Japan; ^3^Cardiovascular Stroke Renal Project (CRP), 654-0142 Hyogo, Japan; ^4^Department of Sport and Medical Science, Faculty of Medical Technology, Teikyo University, 192-0395 Tokyo, Japan; ^5^Department of Cardiology, Sanda City Hospital, 669-1321 Hyogo, Japan

**Keywords:** peak oxygen uptake, heart disease, renal dysfunction, end-tidal oxygen partial pressure

## Abstract

**Background:**

Identifying the causes of low peak oxygen uptake (peak V̇$O_{2}$) in heart disease patients with renal dysfunction is necessary for prognostic improvement strategies. The purpose of this study was to verify the determinants of peak V̇$O_{2}$ for each stage of renal function in heart disease patients, focusing on end-tidal oxygen partial pressure (${\mathrm{PETO}}_{2}$).

**Methods:**

Two hundred fifty heart disease patients who underwent cardiopulmonary exercise testing (CPET) in our institution were consecutively enrolled. Patients were divided into three groups by their estimated glomerular filtration rate (eGFR): <45, 45–59 and ≥60 mL/min/1.73 $m^{2}$. Patient characteristics and CPET parameters including Δ_2_ (rest—anaerobic threshold) were compared between the groups. The relationship between ΔPETO${\mathrm{PETO}}_{2}$ and peak V̇$O_{2}$ was also investigated for each group.

**Results:**

In total, 201 patients were analyzed. Δ${\mathrm{PETO}}_{2}$ decreased with the deterioration of renal function (eGFR <45, 0.1 mmHg vs. eGFR 45–59, 2.4 mmHg vs. eGFR ≥60, 5.2 mmHg, *p *< 0.001). In the eGFR <45 group, left ventricular ejection fraction (LVEF) and hemoglobin (Hb) were significantly associated with peak V̇$O_{2}$β = 0.518, *p *< 0.001 and β = 0.567, *p *< 0.001, respectively), whereas Δ${\mathrm{PETO}}_{2}$ was not. In the eGFR 45–59 group, age, Hb, and Δ${\mathrm{PETO}}_{2}$ showed a significant association with peak V̇$O_{2}$ (β = –0.354, *p* = 0.006; β = 0.258, *p* = 0.007; β = 0.501, *p *< 0.001; respectively). In the univariate analysis, eGFR 45–59 group showed the highest coefficient of determination of Δ${\mathrm{PETO}}_{2}$ to peak V̇$O_{2}$ (*$R^{2}$* = 0.247, *p *< 0.001).

**Conclusions:**

The determinants of peak V̇$O_{2}$ in heart disease patients depended on the stage of renal function. The determinants of peak V̇$O_{2}$ in patients with eGFR <45 were LVEF and Hb, while Δ${\mathrm{PETO}}_{2}$ was the strongest predictor of peak V̇$O_{2}$ in patients with eGFR 45–59.

## 1. Introduction

The prevalence of chronic kidney disease (CKD) is increasing steadily around the world, and a “CKD epidemic” is being warned against [1]. As well, the rate of complications from renal dysfunction in patients with heart disease is also rising. In recent reports, the proportion of patients with renal dysfunction was 48% for those with coronary artery disease [2], 41% for heart failure with reduced ejection fraction [3], and 51% for heart failure with preserved ejection fraction [4]. In fact, about half of all heart disease patients have renal dysfunction. These patients have lower peak oxygen uptake (peak V̇$O_{2}$) [5], and it decreases as renal dysfunction progresses [6]. Lower peak V̇$O_{2}$ is a serious problem in this cohort as it is a predictor of cardiovascular events and mortality [6, 7, 8]. To improve peak V̇$O_{2}$, it is necessary to verify the cause of the low peak V̇$O_{2}$ and take appropriate countermeasures. However, the factors that influence low peak V̇$O_{2}$ in heart disease patients are diverse [9], and the addition of renal dysfunction further complicates the search for causative factors [10]. This problem cannot be overlooked in improving the prognosis of heart disease patients with renal dysfunction. Since the pathophysiology of renal dysfunction and cardiorenal syndrome differs depending on the stage of renal dysfunction [11], it is necessary to verify the determinants of peak V̇$O_{2}$ in heart disease patients by stage of renal dysfunction. On the basis of the above, we hypothesized that the determinants of peak V̇$O_{2}$ in heart disease patients with renal dysfunction depend on the stage of renal dysfunction. The determinants of peak V̇$O_{2}$ are dividing into the oxygen delivery and oxygen extraction [9, 10]. It has been clarified that the contributions of oxygen extraction are greater than those of oxygen delivery in CKD patients [12]. Therefore, in this study, we focused on end-tidal oxygen partial pressure (${\mathrm{PETO}}_{2}$), which has been reported to be associated with renal dysfunction and to show oxygen extraction capacity in skeletal muscle [13, 14, 15, 16]. The purpose of this study was to verify the determinants of peak V̇$O_{2}$ for each stage of renal function in heart disease patients, including ${\mathrm{PETO}}_{2}$.

## 2. Methods

### 2.1 Study Design and Patients

This was a retrospective, single-center, observational study. From April 2016 to August 2021, 250 patients with heart disease (defined as myocardial infarction, angina, and chronic heart failure) who underwent cardiopulmonary exercise testing (CPET) in our institution were consecutively enrolled in the study. Exclusion criteria included patients with a resting respiratory exchange ratio (RER) ≥1.00 due to resting hyperventilation and abnormal breathing [17] and peak RER <1.10 during CPET [18], AT impossible to determine, and no laboratory data measured during CPET. Patients’ characteristics and clinical parameters including age, sex, body mass index, left ventricular ejection fraction (LVEF), medical history, laboratory values during CPET (estimated glomerular filtration rate [eGFR (mL/min/1.73 $m^{2}$)], hemoglobin [Hb (g/dL)]), medications, and the results of CPET were obtained from the electronic medical records by two physical therapists. Laboratory values at CPET were extracted within 2 weeks around the date of CPET.

### 2.2 Definition

eGFR in this study was evaluated with the Japanese version of the following equation: eGFR = 194 × (serum creatinine) – 1.094 × age – 0.287 (× 0.739 if female) [19].

### 2.3 Cardiopulmonary Exercise Testing

All patients underwent symptom-limited maximal CPET using a cycle ergometer (Strength Ergo 8; Mitsubishi Electric Engineering Co., Ltd., Tokyo, Japan) with a 10 watt/min continuous ramp exercise protocol after an initial 3-min rest period and a 4-min warm-up period. The warm-up wattage was chosen to be 0 watts or 20 watts in consideration of age, sex, cardiac function, and exercise habits. During CPET, analysis of expired gas was performed with an AE-310S analyzer (Minato Medical Science, Osaka, Japan). The patients were encouraged to perform a maximal or near maximal effort by monitoring the RER at ≥1.10 [18]. Peak V̇$O_{2}$was defined as the mean value of V̇$O_{2}$ during the last 15 s of the test, and %peak V̇$O_{2}$ was also calculated. AT was determined using the V-slope, ventilatory equivalents, and end-tidal pressure methods based on the statement from the American Heart Association [17] by at least two experts in CPET. Resting ${\mathrm{PETO}}_{2}$ was determined as the mean value during the last 30 s of the rest, and AT ${\mathrm{PETO}}_{2}$ was the ${\mathrm{PETO}}_{2}$ at AT. Δ${\mathrm{PETO}}_{2}$ was the difference between the resting ${\mathrm{PETO}}_{2}$ and AT ${\mathrm{PETO}}_{2}$. Peak oxygen pulse (peak $O_{2}$ pulse), minute ventilation-carbon dioxide production linear regression slope (V̇E vs. V̇${\mathrm{CO}}_{2}$ slope), and minimum ventilatory equivalent for carbon dioxide (V̇E/V̇${\mathrm{CO}}_{2}$) were also obtained. Peak work rate was defined as the work rate at peak V̇$O_{2}$.

### 2.4 Statistical Analysis

Patients were stratified according to their eGFR into three clinically meaningful strata: <45, 45–59, and ≥60 mL/min/1.73 $m^{2}$ [20]. Data are expressed as mean values ± standard deviation (SD) or median (interquartile range) for continuous variables, as appropriate. Normality of distribution was verified using the Shapiro-Wilk test. Categorical variables are presented as numbers and percentages. One-way ANOVA test and the Kruskal-Wallis test were used for comparison between groups, and the χ^2^ test and Fisher’s exact test were used for comparing categorical variables. We used the Bonferroni test as post hoc test. Multivariate linear regression analysis was performed to evaluate independent determinants of peak V̇$O_{2}$ after adjusting for all significant determinants on univariate linear regression analyses. In addition, resting ${\mathrm{PETO}}_{2}$ was also included as a confounding factor to rule out the effect of resting ${\mathrm{PETO}}_{2}$ on Δ${\mathrm{PETO}}_{2}$. Univariate linear regression analyses were performed to evaluate the contribution of each determinant to peak V̇$O_{2}$. A *p*-value of <0.05 was considered to indicate statistical significance. The statistical analyses were performed with EZR (Saitama Medical Center, Jichi Medical University, Saitama, Japan), which is a graphical user interface for R (The R Foundation for Statistical Computing, Vienna, Austria).

## 3. Results

**Fig. 1.**
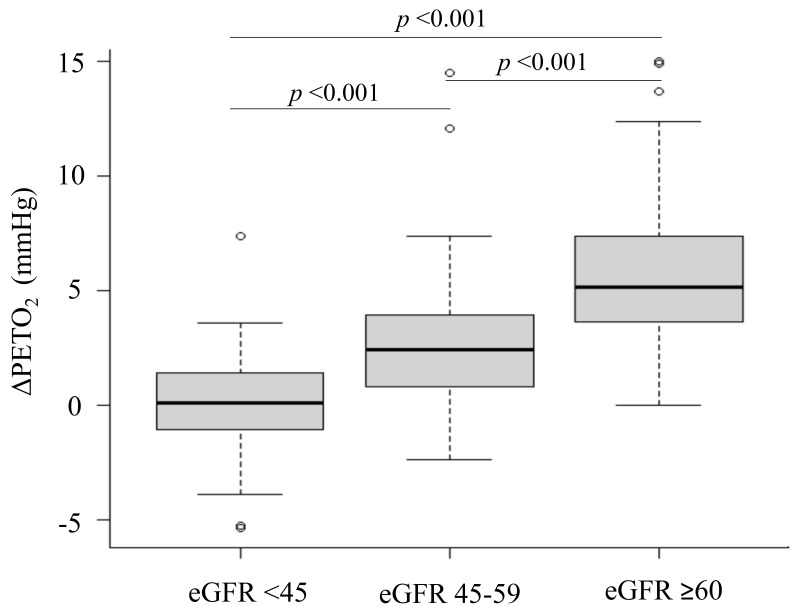
**Comparison of the change in end-tidal oxygen partial pressure (Δ${\mathrm{PETO}}_{2}$) at different values of estimated glomerular filtration rate (eGFR)**.

Of the 250 heart disease patients who underwent CPET, 49 patients were excluded because of rest RER ≥1.00 (n = 6), peak RER <1.10, judgement of AT impossible (n = 8), and no laboratory data (n = 4). Finally, 201 patients were enrolled in the analysis. All patients were divided into three groups by eGFR level: eGFR <45 group (n = 30, 14.9%), eGFR 45–59 (n = 59, 29.4%), and eGFR ≥60 group (n = 112, 55.7%). Table 1 shows the clinical characteristics and CPET parameters of the three groups. The patients in the eGFR <45 group were older and had a higher proportion of chronic heart failure and lower LVEF and Hb. There was a significant difference in peak V̇$O_{2}$ between the three groups (eGFR <45, 16.2 ± 3.9 mL/min/kg vs. eGFR 45–59, 19.7 ± 4.7 mL/min/kg vs. eGFR ≥60, 23.0 ± 4.5 mL/min/kg, *p *< 0.002). Δ${\mathrm{PETO}}_{2}$ decreased with the deterioration of renal function (eGFR <45, 0.1 mmHg vs. eGFR 45–59, 2.4 mmHg vs. eGFR ≥60, 5.2 mmHg, *p *< 0.001) (Fig. 1). There was no significant difference in peak RER and rest ${\mathrm{PETO}}_{2}$ between the three groups.

**Table 1.** **Patient characteristics and CPET parameters**.

		eGFR <45	eGFR 45–59	eGFR ≥60	*p*-value		
		(n = 30)	(n = 59)	(n = 112)	eGFR <45 vs. eGFR 45–59	eGFR 45–59 vs. eGFR ≥60	eGFR <45 vs. eGFR ≥60
Age, years		71.4 ± 7.7	67.8 ± 7.8	61.2 ± 10.8	0.300	<0.001	<0.0001
Male, n (%)		29 (96.7)	50 (84.7)	104 (92.9)	0.46	0.33	1
Body mass index, kg/$m^{2}$		23.2 ± 2.8	23.1 ± 3.2	23.8 ± 2.9	1	0.45	0.88
MI, n (%)		19 (63.3)	43 (72.9)	86 (76.8)	1	1	0.63
AP, n (%)		0 (0)	4 (6.8)	16 (14.3)	0.887	0.632	0.071
CHF, n (%)		24 (80.0)	19 (32.2)	25 (22.3)	<0.001	0.666	<0.001
LVEF, (%)		51.2 (38.9–54.4)	58.7 (49.0–65.4)	59.3 (51.5–68.2)	0.090	0.384	<0.001
Hypertension, n (%)		24 (80.0)	36 (61.0)	74 (66.1)	0.35	1	0.64
Diabetes, n (%)		17 (56.7)	20 (33.9)	36 (32.1)	0.201	1	0.073
Laboratory values							
	eGFR, mL/min/1.73 $m^{2}$	36.8 (32.2–40.5)	54.6 (51.7–57.0)	71.2 (65.2–80.0)	<0.001	<0.001	<0.001
	Hemoglobin, g/dL	12.7 ± 1.8	13.3 ± 1.5	14.2 ± 1.3	0.221	<0.001	<0.001
Medications							
	Beta blockers, n (%)	23 (76.7)	45 (77.6)	72 (64.9)	1	0.38	0.95
	ACE-I, n (%)	8 (26.7)	9 (15.3)	33 (29.5)	0.94	0.19	1
	ARB, n (%)	15 (50.0)	24 (40.7)	39 (34.8)	1	1	0.57
	CCB, n (%)	9 (30.0)	6 (10.2)	21 (18.8)	0.12	0.64	0.83
	Diuretics, n (%)	19 (63.3)	16 (27.1)	14 (12.5)	0.006	0.088	<0.001
	Statin, n (%)	19 (63.3)	49 (83.1)	95 (84.8)	0.212	1	0.054
CPET parameters							
	Peak V̇$O_{2}$, mL/min/kg	16.2 ± 3.9	19.7 ± 4.7	23.0 ± 4.5	0.002	<0.001	<0.001
	%Peak V̇$O_{2}$, %	70.6 ± 16.4	82.8 ± 18.7	92.8 ± 19.0	0.011	0.003	<0.001
	AT V̇$O_{2}$, mL/min/kg	10.9 ± 2.1	12.4 ± 2.5	14.0 ± 2.6	0.029	<0.001	<0.001
	Peak RER	1.20 ± 0.05	1.20 ± 0.06	1.18 ± 0.06	1	0.086	0.382
	AT RER	0.96 ± 0.02	0.96 ± 0.03	0.95 ± 0.04	1	0.14	0.11
	Peak WR, watts	86.2 ± 17.2	102.3 ± 28.6	122.9 ± 28.3	0.028	<0.001	<0.001
	V̇E vs. V̇${\mathrm{CO}}_{2}$ slope	33.9 (30.8–38.5)	30.6 (27.9–33.5)	29.2 (26.3–31.7)	0.007	0.137	<0.001
	Minimum V̇E/V̇${\mathrm{CO}}_{2}$	36.1 (33.5–39.9)	33.9 (30.9–37.4)	30.8 (28.8–34.5)	0.173	0.002	<0.001
	Peak $O_{2}$ pulse	8.6 ± 2.0	9.6 ± 2.5	11.1 ± 2.2	0.181	<0.001	<0.001
	ΔV̇$O_{2}$/ΔWR	8.1 ± 1.6	8.8 ± 1.4	9.4 ± 1.3	0.051	0.030	<0.001
	Rest ${\mathrm{PETO}}_{2}$, mmHg	107.2 ± 5.5	107.8 ± 4.9	108.1 ± 4.2	1	1	0.88
	AT ${\mathrm{PETO}}_{2}$, mmHg	107.1 ± 5.4	105.0 ± 5.5	102.4 ± 4.7	0.216	0.004	<0.001
	Δ${\mathrm{PETO}}_{2}$, mmHg	0.1 (–1.1–1.4)	2.4 (0.8–4.0)	5.2 (3.7–7.4)	<0.001	<0.001	<0.001

CPET, cardiopulmonary exercise testing; eGFR, estimated glomerular filtration rate; MI, myocardial infarction; AP, angina pectoris; CHF, chronic heart failure; LVEF, left ventricular ejection fraction; ACE-I, angiotensin converting enzyme inhibitor; ARB, angiotensin II receptor blocker; CCB, calcium channel blocker; V̇$O_{2}$, oxygen uptake; AT, anaerobic threshold; RER, respiratory exchange ratio; WR, work rate; V̇E, expiratory minute volume; V̇${\mathrm{CO}}_{2}$, carbon dioxide output; V̇E/V̇${\mathrm{CO}}_{2}$, ventilatory equivalent for carbon dioxide; $O_{2}$, oxygen; ${\mathrm{PETO}}_{2}$, end-tidal oxygen partial pressure. Values shown are % (n), mean ± standard deviation, or median (interquartile range).

The results of univariate and multivariate linear regression analysis in all subjects showed that age (β = –0.142, *p* = 0.023), LVEF (β = 0.150, *p* = 0.006), eGFR strata (β = 0.154, *p* = 0.026), Hb (β = 0.167, *p* = 0.005), and Δ${\mathrm{PETO}}_{2}$ (β = 0.356, *p *< 0.001) were significantly associated with peak V̇$O_{2}$ (Table 2).

**Table 2.** **Univariate and multivariate linear regression analyses for peak V̇$O_{2}$ in all subjects**.

	Univariate		Multivariate		
	*β*	*p*-value	*β*	95% CI	*p*-value
Age	–0.451	<0.001	–0.142	–0.128, –0.009	0.023
LVEF	0.113	<0.001	0.150	0.018, 0.110	0.006
eGFR strata	0.508	<0.001	0.154	0.128, 2.000	0.026
Hb	0.148	<0.001	0.167	0.164, 0.914	0.005
Δ${\mathrm{PETO}}_{2}$	0.552	<0.001	0.356	0.308, 0.690	<0.001
Rest Δ${\mathrm{PETO}}_{2}$	0.121	0.020	–0.194	–0.335, –0.092	<0.001
* $R^{2}$ *					*0.462*

V̇$O_{2}$, oxygen uptake; CI, confidence interval; LVEF, left ventricular ejection fraction; eGFR, estimated glomerular filtration rate; Hb, hemoglobin; ${\mathrm{PETO}}_{2}$, end-tidal oxygen partial pressure.

The results of univariate and multivariate linear regression analyses differed between the eGFR strata. In the eGFR <45 group, LVEF and Hb were significantly associated with peak V̇$O_{2}$ (β = 0.518, *p <* 0.001 and β = 0.567, *p <* 0.001, respectively). In the eGFR 45–59 group, age, Hb, and Δ${\mathrm{PETO}}_{2}$ showed a significant association with peak V̇$O_{2}$ (β = –0.354, *p *= 0.006; β = 0.258, *p *= 0.007; β = 0.501,* p <* 0.001; respectively). In the eGFR ≥60 group, Δ${\mathrm{PETO}}_{2}$ was significantly associated with peak V̇$O_{2}$ (β = 0.308, *p *= 0.003) (Table 3).

**Table 3.** **Univariate and multivariate linear regression analyses for peak V̇$O_{2}$ by eGFR strata**.

		Univariate		Multivariate		
		β	*p*-value	β	95% CI	*p*-value
eGFR <45 group						
	Age	–0.358	0.052			
	LVEF	0.572	<0.001	0.518	0.086, 0.229	<0.001
	Hb	0.616	<0.001	0.567	0.728, 1.766	<0.001
	Δ${\mathrm{PETO}}_{2}$	0.175	0.356			
	Rest Δ${\mathrm{PETO}}_{2}$	0.121	0.059			
* $R^{2}$ *						*0.620*
eGFR 45–59 group						
	Age	–0.521	<0.001	–0.354	–0.297, –0.052	0.006
	LVEF	0.183	0.166			
	Hb	0.365	0.004	0.258	0.241, 1.449	0.007
	Δ${\mathrm{PETO}}_{2}$	0.523	<0.001	0.501	0.402, 1.013	<0.001
	Rest Δ${\mathrm{PETO}}_{2}$	0.062	0.058	–0.181	–0.384, –0.016	0.035
* $R^{2}$ *						*0.538*
eGFR ≥60 group						
	Age	–0.236	0.012	–0.215	–0.140, 0.017	0.125
	LVEF	0.198	0.036	0.146	–0.014, 0.137	0.113
	Hb	0.078	0.416			
	Δ${\mathrm{PETO}}_{2}$	0.314	<0.001	0.308	0.154, 0.716	0.003
	Rest Δ${\mathrm{PETO}}_{2}$	0.036	0.045	–0.193	–0.154, –0.013	0.037
* $R^{2}$ *						*0.194*

V̇$O_{2}$, oxygen uptake; CI, confidence interval; eGFR, estimated glomerular filtration rate; LVEF, left ventricular ejection fraction; Hb, hemoglobin; ${\mathrm{PETO}}_{2}$, end-tidal oxygen partial pressure.

**Fig. 2.**
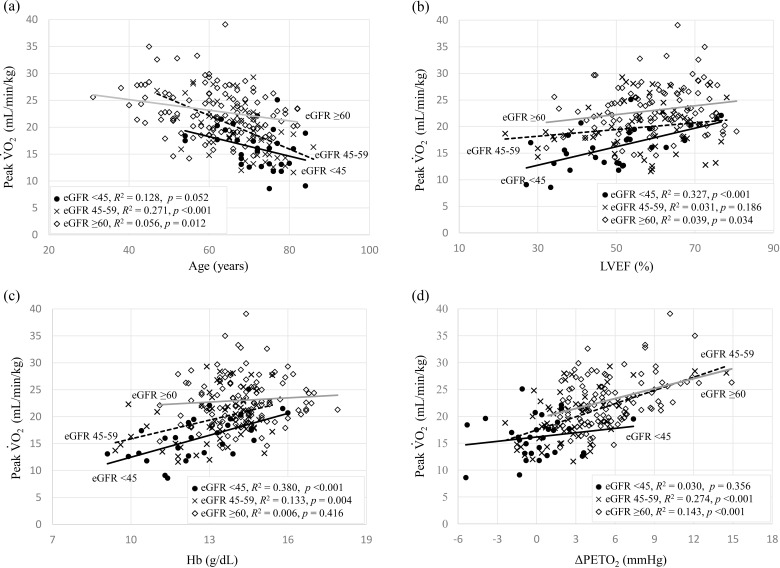
**Coefficients of determination for peak V̇$O_{2}$ for each group**. Coefficients of determination of the (a) age, (b) left ventricular ejection fraction (LVEF), (c) hemoglobin (Hb), and (d) change in end-tidal oxygen partial pressure (Δ${\mathrm{PETO}}_{2}$) to peak V̇$O_{2}$ for each group.

Fig. 2 summarizes the coefficients of determination of age, LVEF, Hb, and Δ${\mathrm{PETO}}_{2}$ for peak V̇$O_{2}$ by eGFR level. In the eGFR 45–59 group, the coefficient of determination for peak V̇$O_{2}$ was higher in age and Δ${\mathrm{PETO}}_{2}$ than in the other groups (*$R^{2}$* = 0.241, *p *< 0.001; *$R^{2}$* = 0.247, *p *< 0.001; respectively). The eGFR <45 group showed higher coefficients of determination for peak V̇$O_{2}$ in LVEF and Hb than in the other groups (*$R^{2}$* = 0.327, *p *< 0.001; *$R^{2}$* = 0.380, *p *< 0.001; respectively). The *p* value for interaction analysis of the slope difference was <0.001.

## 4. Discussion

This study revealed that the determinants of peak V̇$O_{2}$ depend on the stage of renal function in heart disease patients. In the group with eGFR <45, the determinants of peak V̇$O_{2}$ were LVEF and Hb. In the group with eGFR 45–59, Δ${\mathrm{PETO}}_{2}$ was the most influential determinant of peak V̇$O_{2}$. As a result of examining the determinants of peak V̇$O_{2}$ in all subjects, age, LVEF, Hb, and Δ${\mathrm{PETO}}_{2}$ were all determinants independently of eGFR strata. Thus, the present study clarified that in patients with heart disease with renal dysfunction, it is necessary to investigate the determinants for each level of renal dysfunction. The decrease in peak V̇$O_{2}$ is observed in the stage of mild renal dysfunction [21]. In this study as well, peak V̇$O_{2}$ was significantly decreased even in the eGFR 45–59 group. In this group, age, Hb and Δ${\mathrm{PETO}}_{2}$ were the determinants of peak V̇$O_{2}$, and both oxygen delivery capacity and oxygen extraction capacity affected peak V̇$O_{2}$ in this group. Among these factors, multivariate analysis showed that Δ${\mathrm{PETO}}_{2}$ had the highest β for peak V̇$O_{2}$. Furthermore, in the univariate analysis, this group showed the highest contribution of Δ${\mathrm{PETO}}_{2}$ to peak V̇$O_{2}$.

${\mathrm{PETO}}_{2}$ reflects the oxygen extraction capacity of skeletal muscle during incremental exercise up to AT [13, 14], it is also reported to reflect mitochondrial oxygen uptake [15]. Although the subjects of these prior studies were mainly healthy individuals, in our study of patients with myocardial infarction, ${\mathrm{PETO}}_{2}$ at AT was affected by abnormal ventilation, whereas Δ${\mathrm{PETO}}_{2}$ from rest to AT reflected peripheral factors of peak V̇$O_{2}$ [16].

Therefore, it is highly possible that Δ${\mathrm{PETO}}_{2}$ represents the oxygen extraction capacity of skeletal muscle, that is, mitochondrial function. This study showed that Δ${\mathrm{PETO}}_{2}$ decreased as renal dysfunction progressed.

Heart disease patients have decreased mitochondrial function due to oxidative stress, inflammation, and insulin resistance due to heart disease [22, 23]. In addition, heart disease risk factors such as hyperglycemia, hyperlipidemia, and smoking also reduce mitochondrial function [24]. With the addition of renal dysfunction in these patients, oxidative stress, inflammation, and uremic toxins from the renal dysfunction cause further mitochondrial dysfunction [25, 26, 27]. Furthermore, in heart failure patients with renal dysfunction, the relation between the cardiac and renal dysfunction of cardiorenal syndrome, which adversely affect each other [11], may contribute to a further decline in mitochondrial function. A report that mitochondrial dysfunction worsens as renal dysfunction progresses also supports this result [28].

The most interesting finding in this study was that although Δ${\mathrm{PETO}}_{2}$, which represents oxygen extraction capacity, decreased as renal dysfunction progressed, Δ${\mathrm{PETO}}_{2}$ was not a determinant of peak V̇$O_{2}$ in the eGFR <45 group. As peak V̇$O_{2}$ is composed of the product of oxygen delivery capacity times oxygen extraction capacity, there is no doubt that a decrease in oxygen extraction capacity will lead to a decrease in peak V̇$O_{2}$. However, in this study, the determinants of peak V̇$O_{2}$ in the eGFR <45 group were LVEF and Hb, which are mainly related to oxygen delivery capacity. In this regard, as the eGFR <45 group had significantly lower LVEF and Hb than the other two groups, decreased oxygen delivery capacity may be the main contributor to the decrease in peak V̇$O_{2}$. A previous study also reported that LVEF is not a determinant of peak V̇$O_{2}$ [29]. However, LVEF in the present study was the determinant in heart disease patients with moderate to severe renal dysfunction. The mechanism for this is unknown, but it may be a characteristic of heart disease patients with an eGFR <45. This result also contrasted with recent reports that low oxygen extraction capacity is the main factor for low peak V̇$O_{2}$ in CKD patients [5, 12]. The Δ${\mathrm{PETO}}_{2}$ of the eGFR <45 group was very low at 0.1 mmHg, and it is estimated that oxygen extraction of skeletal muscle would hardly increase during incremental exercise in these patients. As skeletal muscle oxygen extraction cannot be increased, it may be necessary for these patients to rely on oxygen delivery to increase oxygen uptake. Thus, this may be the reason why the only determinants of peak V̇$O_{2}$ were the factors related to oxygen delivery capacity. This also supports the finding that the contribution of LVEF and Hb to peak V̇$O_{2}$ in this group was higher than that in the other groups. The effects of exercise training aimed at improving mitochondrial function and oxygen extraction capacity to improve peak V̇$O_{2}$ have been reported [30], but in heart disease patients with eGFR <45, such interventions may not lead to improvement in peak V̇$O_{2}$. Improving oxygen delivery capacity may be more important. Further verification is needed on interventions to improve peak V̇$O_{2}$ in this group.

Heart disease patients with eGFR <45 experience increased cardiovascular events [31]. One of the causes is suggested to be that cardiac load is increased due to the abnormally low value of oxygen extraction capacity being compensated for by oxygen delivery capacity.

Regarding the clinical implication of this study, the first was that decrease in Δ${\mathrm{PETO}}_{2}$ with the progression of renal dysfunction revealed that the oxygen extraction capacity of skeletal muscle decreased as renal dysfunction progressed. Second, in the eGFR <45 group, Δ${\mathrm{PETO}}_{2}$ was not a determinant, and the determinant of peak V̇$O_{2}$ was different depending on the degree of renal dysfunction. Therefore, intervention strategies for improving peak V̇$O_{2}$ in heart disease patients should be considered for each stage of renal dysfunction. The effects of exercise training aimed at improving mitochondrial function and oxygen extraction capacity to improve peak V̇$O_{2}$ have been reported [31], but in heart disease patients with eGFR <45, such interventions may not lead to improvement in peak V̇$O_{2}$. Improving oxygen delivery capacity may be more important. A meta-analysis has been reported that Fe therapy improved peak V̇$O_{2}$ in patients with heart failure with reduced EF [32]. Further verification is needed on interventions to improve peak V̇$O_{2}$ in this group. On the other hand, in heart disease patients with eGFR 45–59, interventions that improve skeletal muscle oxygen extraction, i.e., mitochondrial function, may be effective. In recent years, it has been reported that exercise improves mitochondrial function in heart disease patients [33, 34].

### Study Limitations

This study has several limitations. First, this was a single-center, retrospective study consisting of a relatively small number of patients. Second, there is potential selection bias as patients who were unable to undergo CPET due to frailty and sarcopenia were excluded. Because these factors themselves are associated with mitochondrial dysfunction [35, 36], further investigation of these patients is needed. Third, eGFR calculated with serum creatinine is affected by skeletal muscle mass and may not accurately reflect renal function [37]. Fourth, renal dysfunction is classified into acute kidney injury, CKD, and worsening renal function [38]. Further studies are needed to determine whether each clinical status may have different effects on skeletal muscle oxygen extraction capacity. Fifth, there was a significant difference in the etiology between the groups. Future studies will need to be validated for association with etiology. Finally, we could not evaluate cardiac output, vascular function including that of the capillaries, and skeletal muscle mass, which are additional determinants of peak V̇$O_{2}$.

## 5. Conclusions

Δ${\mathrm{PETO}}_{2}$, which indicates the oxygen extraction capacity of skeletal muscle, decreased with the progression of renal dysfunction. In the eGFR 45–59 group, Δ${\mathrm{PETO}}_{2}$ was the strongest determinant of peak V̇$O_{2}$, but the determinants in the eGFR <45 group were LVEF and Hb, and Δ${\mathrm{PETO}}_{2}$ was not included. This study suggests that intervention strategies should be considered for each stage of renal dysfunction to improve peak V̇$O_{2}$ in heart disease patients.

## Abbreviations

CKD, chronic kidney disease; CPET, cardiopulmonary exercise testing; eGFR, estimated glomerular filtration rate; LVEF, left ventricular ejection fraction; peak V̇$O_{2}$, peak oxygen uptake; ${\mathrm{PETO}}_{2}$, end-tidal oxygen partial pressure; RER, respiratory exchange ratio; V̇${\mathrm{CO}}_{2}$, carbon dioxide production; V̇E, minute ventilation.
